# Impaired immunosuppressive effect of bone marrow mesenchymal stem cell-derived exosomes on T cells in aplastic anemia

**DOI:** 10.1186/s13287-023-03496-0

**Published:** 2023-10-04

**Authors:** Shichong Wang, Jiali Huo, Yilin Liu, Lingyun Chen, Xiang Ren, Xingxin Li, Min Wang, Peng Jin, Jinbo Huang, Neng Nie, Jing Zhang, Yingqi Shao, Meili Ge, Yizhou Zheng

**Affiliations:** 1 Diagnostic and Therapeutic Center for Anemic Diseases, State Key Laboratory of Experimental Hematology, National Clinical Research Center for Blood Diseases, Haihe Laboratory of Cell Ecosystem, Institute of Hematology & Blood Diseases Hospital, Chinese Academy of Medical Sciences & Peking Union Medical College, Tianjin, 300020 China; 2 Department of Hematology, Shanghai Ninth People’s Hospital, Shanghai Jiao Tong University School of Medicine, Shanghai, 200011 China

**Keywords:** Acquired aplastic anemia, Exosome, microRNA, Immunoregulation

## Abstract

**Background:**

Previous studies have verified the dysfunction of mesenchymal stem cells (MSCs) for immunoregulation in acquired aplastic anemia (AA) patients. Exosomes derived from MSCs can partially substitute MSCs acting as immune regulator. Dysfunction of exosomes (Exos) derived from AA-MSC (AA-Exos) may play a key role in immunologic dissonance.

**Method:**

In this study, CD3 + T cells were collected and cocultured with AA-Exos and exosomes derived from HD-MSC (HD-Exos). The proliferation, differentiation and activation of CD3 + T cells were detected to compare the immunosuppressive effects between AA-Exos and HD-Exos. An immune-mediated murine model of AA was structured to compare the therapeutic effect of AA-Exos and HD-Exos. Furthermore, total RNA including miRNA from exosomes we purified and total RNA of CD3 + T cells were extracted for RNA-seq in order to construct the miRNA–mRNA network for interactions and functional analysis.

**Results:**

AA-Exos had impaired inhibition effects on CD3 + T cells in terms of cell proliferation, activation and differentiation compared with exosomes from HD-Exos. HD-Exos showed a more effective rescue of AA mice compared to AA-Exos. Importantly, we found some differentially expressed miRNA involved in immune response, such as miR-199, miR-128 and miR-486. The Gene Ontology analysis of differentially expressed genes (DEGs) revealed involvement of various cellular processes, such as lymphocyte chemotaxis, lymphocyte migration and response to interferon-gamma. The Kyoto Encyclopedia of Genes and Genomes analysis illustrated upregulation of critical pathways associated with T cell function after coculturing with AA-Exos compared with HD-Exos, such as graft-versus-host disease, Th17 cell differentiation and JAK-STAT signaling pathway. A miRNA–mRNA network was established to visualize the interaction between them.

**Conclusion:**

In summary, AA-Exos had impaired immunosuppressive effect on T cells, less ability to rescue AA mice and differently expressed miRNA profile, which might partly account for the pathogenesis of AA as well as provide a new target of AA treatment.

**Supplementary Information:**

The online version contains supplementary material available at 10.1186/s13287-023-03496-0.

## Introduction

Aplastic anemia (AA) is a disease characterized by pancytopenia, with leukocytopenia, thrombopenia and anemia, caused by diverse pathogenic mechanisms, with the final outcome of hematopoietic failure. Seventy percent of patients with acquired aplastic anemia were attributed to immunity dysfunction due to the positive response to the immunosuppressive therapy [1]. In addition, it was confirmed that functionally and phenotypically activated T cells were involved in the pathophysiology of the AA [2, 3]. Therefore, the critical aspect of AA treatment depended on understanding the causes of immune dysregulation and how to reshape immune microenvironment, especially for refractory AA.

Mesenchymal stem cells (MSCs) have gained much interest owing to self-renewal capacity, multilineage differentiation potential and immunosuppressive property, which was demonstrated as a promising strategy for regenerative medicine [4, 5]. Previous studies unveiled that MSCs mainly functioned through paracrine secretion because vast majority of them were trapped in pulmonary tissue in transplantation [6]. So it was reasonable that functional growth factors, exosomes and chemokines released by MSCs in situ could be a mechanism by which MSCs contributed to immune suppression in distant sites [7]. Exosome, a subtype of extracellular vesicles, with diameter of 30–150 nm, is formed via the inward budding of the cell plasma membrane with sufficient noncoding RNAs (ncRNAs) and proteins [8]. Several studies revealed that exosomes served as constitutive regulators of cell and tissue homeostasis, which can partially substitute MSC producing an immunosuppressive effect [9, 10]. Dysfunction and pathophysiology of MSCs during acquired AA had been identified [5]. However, differences in the bio function and molecule complexity between AA-MSC-derived exosomes (AA-Exos) and HD-MSC-derived exosomes (HD-Exos) were still unknown. Therefore, it is necessary to conduct a multifaced comparison of them at their function and phenotype level.

In our study, we reviewed the pathophysiological effects of AA-Exos while highlighting on their miRNA expression profile and interactions with T cells compared with HD-Exos.

## Method

### Patients

Blood samples were collected from 35 acquired AA patients and 35 HDs. BM samples were acquired from 34 AA patients and 32 HDs. The characteristics of AA patients and HDs are listed in Additional file 5: Table S1. The diagnosis of AA was according to the criteria of Camitta et al. [11] All patients were newly diagnosed without immunosuppressive therapy (IST) at the time of sample collection. Informed consents were obtained according to the principle of Declaration of Helsinki (Ethics Number: KT2014005-EC-1).

### Isolation of exosomes

Conditioned media from cultures of MSC cells were centrifuged at 300 × g for 10 min to remove any cells or large cellular fragments. Supernatants were then collected and performed using following kits: Qiagen exosome kit, according to the manufacturer’s procedures as previously reported [12]. In brief, supernatants are filtered to exclude particles larger than 0.8 μm, add the sample/XBP mix onto the exoEasy spin column and centrifuge at 500 × *g* for 1 min. Add 10 ml buffer XWP and centrifuge at 5000 × *g* for 5 min to remove residual buffer from the column. Add 400 μl Buffer XE and centrifuge at 500 × *g* for 5 min to collect the eluate. Centrifuge at 5000 × *g* for 5 min to collect the eluate for the following RNA sequencing and experiments.

### Identification of exosomes

For flow cytometry analysis, exosomes were incubated with CD63 and CD81 antibodies and then detected by BD accuri C6 flow cytometer (BD Biosciences, USA). For transmission electron microscopy, exosomes were placed on formvar-coated copper grids overnight. After staining with 1% uranyl acetate for 10 min, samples were observed with TEM operating at 80 kV (Leica, German). To see whether exosomes can be taken up by T cells, exosomes were dyed by PKH76 (Sigma, German) with green fluorescence, and T cells were stained with DAPI (Dojindo, Japan) after 6 h of incubation for confocal microscope imaging (Nikon, Japan).

### The isolation of CD3 + T cells and coculture with MSC-Exos

Peripheral blood mononuclear cells (PBMNCs) were isolated through centrifugation using Ficoll reagent (Haoyang, China). Then CD3 + T cells were positive selected by CD3 microbeads as we previously reported (Miltenyi Biotec, Auburn, USA). For flow cytometry analysis, cells were incubated with fluorochrome-labeled Abs: Fitc CD4, APC CD8, APC/CY7 interferon-γ(IFN-γ), PE interleukin-4 (IL-4) and APC interleukin-17A (IL-17A). The CD3 + T cells were seeded onto 48-well culture plates at a density of 5 * 10^5^ cells/well and cultured with AA/HD MSC-derived exosomes in 500 μl AIM V (Gibco) medium, 5 μl CD3/28beads (Invitrogen, Waltham, USA) were added, 1 μl Cell Stimulation Cocktail (Invitrogen, Waltham, USA) which contained PMA, ionomycin, brefeldin A and monensin was added for 6 h at 37 °C in 5% CO2 before harvest. Then cells were collected to detect intracellular cytokine as described previously. The antibodies involved in this study are listed in Additional file 5: Table S2.

### Murine AA model

All of the animal experiments adhered to the ARRIVE guidelines. Meanwhile, ethical approval of animal research was signed by the Ethical Committee of Institute of Hematology and Blood Diseases Hospital (approval no. KT2017031-EC-2; approval date: 2018-03-28). The C57BL/6 mice, BALB/c mice and (C57BL/6 J × BALB/cBy) F1 (CByB6F1) mice at 8-week-old was obtained from Charles River (Beijing, China). For inducing acute AA mice, lymph node (LN) cells from C57BL/6J mice at a dose of 5*10^6^ per recipient were injected into CByB6F1 mice after a 5 Gy of total body irradiation (TBI) within 4–6 h [13]. Mice with no intervention and mice received irradiation without LN cells injection were divided into normal and TBI groups as controls, respectively. AA mice are divided into 3 groups randomly: AA-Exos group received an injection of 200 μl PBS contained 100 μl exosomes derived from AA-MSC twice a week throughout the treatment until death. HD-Exos group received an injection of 200 μl PBS contained 100 μl exosomes derived from HD-MSC. PBS group received an injection of 200 μl PBS as negative control. Detection of complete blood counts was conducted at 0, 7, 10, 14 after LN cell injection by Sysmex’s flagship analyzer (XN-1000, USA). Animals used for survival observation were returned to appropriate environment following the completion of data collection. Other mice were euthanized by intraperitoneal injection of sodium pentobarbital at a dosage of 150–200 mg/kg. The rationale for using sodium pentobarbital in mice is that it is a potent and fast-acting barbiturate that produces deep anesthesia and death without pain. BM cells, LN cells and spleen cells were extracted for flow cytometry analysis of intracellular cytokines and cell markers as described above. Sternums were obtained for hematoxylin and eosin (H&E) stain. Additional file 5: Table S3 clarified the animal amounts in the experimental design.

### Sequencing analysis of microRNAs and RNAs

Purification of total RNA including miRNA and other noncoding RNAs from exosomes was conducted according to the protocol of exoRNeasy Serum Kit (Qiagen, Hilden, Germany). The small RNAs were mapped to reference sequence by Bowtie [14] to analyze their expression and distribution. The target mRNAs of miRNAs were predicted by MiRanda and RNAhybrid according to the software protocol.

### Quantitative real-time PCR

The verification of miRNA by quantitative real-time PCR was performed by using All-in-One™ miRNA qRT-PCR Detection System kit (Genecopoeia, Guangzhou, China) and QuantStudio 5 system (Applied Biosystems, Carlsbad, CA). The verification of mRNAs was performed using TransScript.

First-Strand cDNA Synthesis Supermix (TransGen Biotech, Beijing, China). The primer sequence of miRNAs and mRNA is listed in Additional file 5: Table S4.

### RNA library construction and sequencing

The total RNA of CD3 + T cells was extracted by TRIzol reagent (Invitrogen, USA) as reported previously. RNA purity was checked using the NanoPhotometer spectrophotometer (IMPLEN, CA, USA). RNA integrity was assessed using the RNA Nano 6000 Assay Kit of the Agilent Bioanalyzer 2100 system (Agilent Technologies, CA, USA). The mRNA was purified from total RNA by using poly-T oligo-attached magnetic beads. Fragmentation was carried out using divalent cations under elevated temperature in First Strand Synthesis Reaction Buffer(5X). To select cDNA fragments of preferentially 370–420 bp in length, the library fragments were purified with the AMPure XP system (Beckman Coulter, Beverly, USA). Then the PCR product was purified by AMPure XP beads, and the library was finally obtained. After the library is qualified, the different libraries are pooled according to the effective concentration and the target amount of data off the machine and then sequenced by the Illumina NovaSeq 6000.

### The miRNA–mRNA network construction and functional analysis

After the RNA library of T cells is constructed, differential expression analysis of two groups was performed using the DESeq2 R package (1.20.0). The resulting *p*-values were adjusted using the Benjamini and Hochberg’s approach for controlling the false discovery rate. We defined the standard of *p* <  = 0.05 and |log2(foldchange)|> = 1 as the threshold for significantly differential expression. Overlap of the predicted target mRNAs of miRNAs and differentially expressed mRNAs of T cells was selected to further analyze the function of the target gene collection. For Gene Ontology (GO) enrichment analysis, we used GOseq-based Wallenius non-central hyper-geometric distribution and was implemented for GO enrichment analysis. For Kyoto Encyclopedia of Genes and Genomes (KEGG) pathway analyses, we used KOBAS software to test the statistical enrichment of the target gene candidates. To determine whether a predefined gene set can show a significant consistent difference between two biological states, we use the GSEA analysis tool http://www.broadinstitute.org/gsea/index.jsp, for GSEA analysis. The networks of miRNA–mRNA interaction were drawn by using Cytoscape3.9.1, and the plug-in of cytoscape named CytoNCA was used to identify the most important microRNA based on degree and closeness.

### Dual-luciferase reporter assay

Cells are transfected with plasmids containing the wild-type (WT) or a mutated (MUT) 3' UTR of TNF mRNA and the mimic of miR-486 or negative control. After lysis, Renilla and firefly luciferase substrates were added. Light emission is measured, and the ratio of firefly/Renilla activity indicates gene expression levels. Three replicate biological samples were assayed each with three technical repeats.

### Statistical analysis

Unpaired t test and one-way ANOVA test were used for analysis of two different unpaired groups and multiple unpaired groups, respectively. All analyses were performed using Prism 9.0 (GraphPad Software, San Diego, CA, USA). *p* < 0.05 was considered as a significant level.

## Result

### Isolation and characterization of MSC exosomes

The isolation process of the exosomes was delineated in a schematic diagram in Additional file 1: Figure S1a. The MSCs from AA patients and HDs were identified as previously reported [5]. Isolated MSC exosomes were identified by various methods including flow cytometry, electron microscopy and nanoparticle tracking analysis (NTA) as commonly required (Additional file 1: Figure S1b-f). Flow cytometry revealed that both AA-Exos and HD-Exos expressed CD81 and CD63 (Additional file 1: Figure S1b), two molecule markers enriched in exosomes. NTA showed that more than 80% of exosomes with the range of the particle size within 20–200 nm (Additional file 1: Figure S1c, d). Transmission electron microscopy (TEM) graphed the spherically shaped vesicles of exosomes (Additional file 1: Figure S1e, f). The bicinchoninic acid assay protein quantification was performed to test and adjust to the same concentration of exosomes for further experiment. To confirm the interaction of exosomes with T cells, we first assessed the ability of the T cells taking up exosomes. Exosomes were dyed by PKH76 with green fluorescence and added to the culture medium (Additional file 1: Figure S1g). After 6 h of incubation, the T cells were harvest and stained with DAPI in shape of cell slide (Additional file 1: Figure S1h). Additional file 1: Figure S1i showed the internalization and localization of PKH‐76 green‐labeled exosomes in the cytoplasm of the T cells. Consequently, all the results proved that exosomes were successfully extracted. Meanwhile, there was no significant difference of the shape, size, or surface markers between AA-Exos and HD-Exos.

### Therapeutic effect of AA-Exos and HD-Exos in treating AA mice

**Fig. 1:**
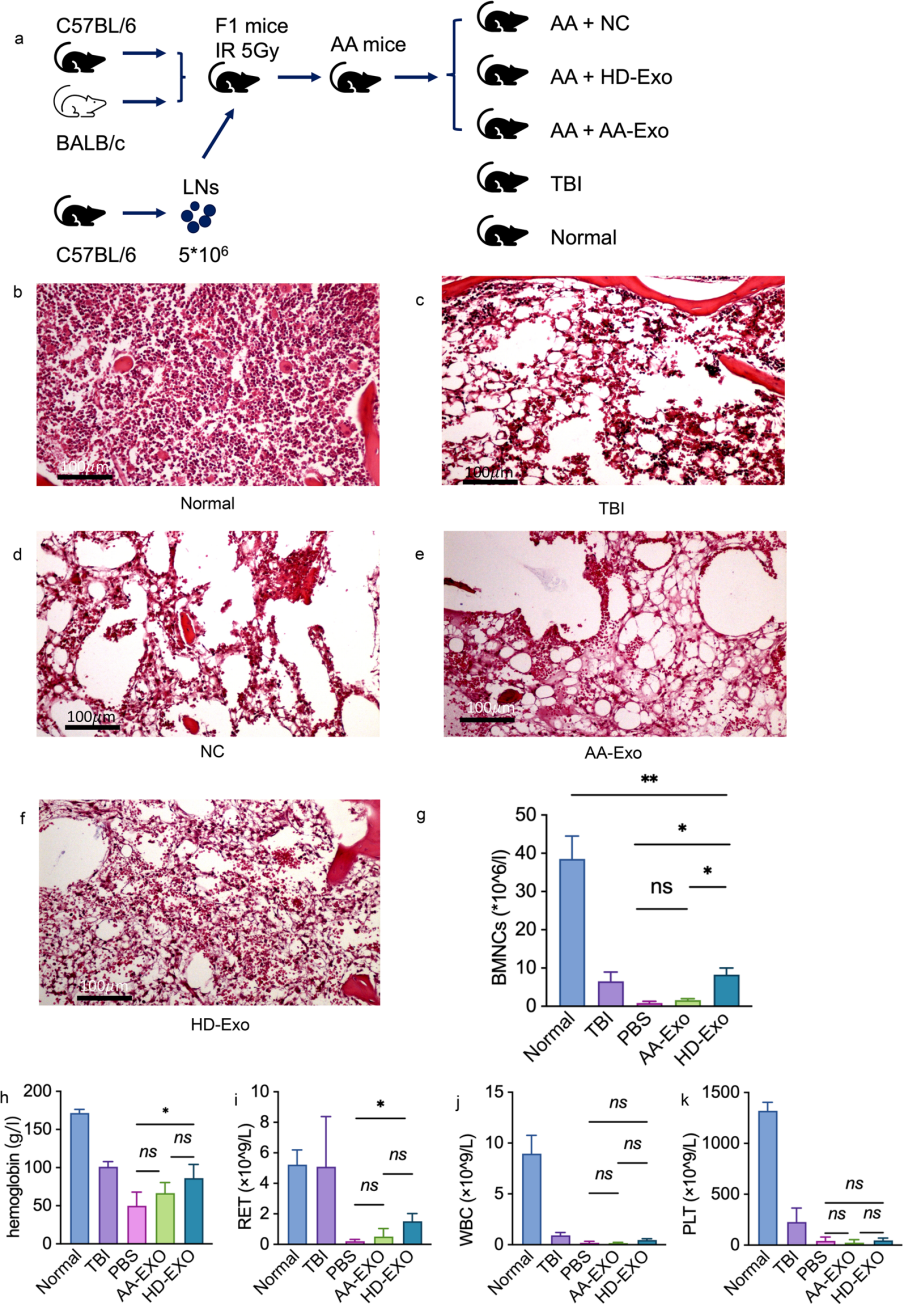
Different therapeutic effect of AA-Exos and HD-Exos in treating AA mice. **a** The schematic diagram of construction of AA mice model. **b**–**f** H&E stain of mice sternum in normal, TBI, PBS, AA-Exo and HD-Exo group. g, BMNC counting of different groups, HD-Exos had more potency in ameliorating bone marrow hyperplasia. **h**–**k** hemoglobulin, RET, WBC and PLT of mice received different treatment on D14, mice received HD-Exos showed improvement in Hb and RET compared with PBS group. (* for 0.01 ≤ *p* < 0.05; ** for *p* < 0.001; *** for *p* < 0.0001, ns for not significant; *n* = 6)

Exosomes were considered as a promising surrogate treatment of MSC insight of their non-cellular alternative [7]. Mohammad et al. [15] had revealed that MSC-exosomes conditionally ameliorated bone marrow failure symptoms in aplastic anemia mice. To compare the therapeutic effect of AA-Exos and HD-Exos, an immune-mediated murine model was structured as reported above [5]. Briefly, C57BL/6 mice were injected with 5*10^6^ LN cells from C57 mice after 4 h exposed to 5 gy irradiation. These mice were randomly divided into three groups for different treatment, with one received PBS injection as negative control, one received HD-Exos and remainder received AA-Exos as treatment which schematically shown in Fig. 1a. Every mouse received injection twice a week until death.

Clinical manifestation was measured by white blood cell (WBC), hemoglobin (Hb), red blood cell (RBC), platelet (PLT), reticulocyte (RET) and nucleated cells count of bone marrow. The first five indexes were measured at D3,7,10,14 by routine analysis of peripheral blood, while BMNC was collected for counting and other flow cytometry analyzing at D14 after the mice were execute to death. Both AA/HD exosomes injection eliminated the damage of the BM, and the bone marrow biopsy staining provides a visualization of the improvement in bone marrow hyperplasia (Fig. 1b–f). The BMNC counting revealed that HD-Exos had more potency in ameliorating bone marrow hyperplasia (Fig. 1g). Mice received HD-Exos showed improvement in Hb and RET compared with negative control (Fig. 1h, i), while no significant increment was observed in AA-Exos. There were no significant differences in WBC and PLT (Fig. 1j, k).

To systematically evaluate the immune states of AA mice, spleen and lymph node were harvested to make single-cell suspension and immune cell populations were analyzed.

**Fig. 2:**
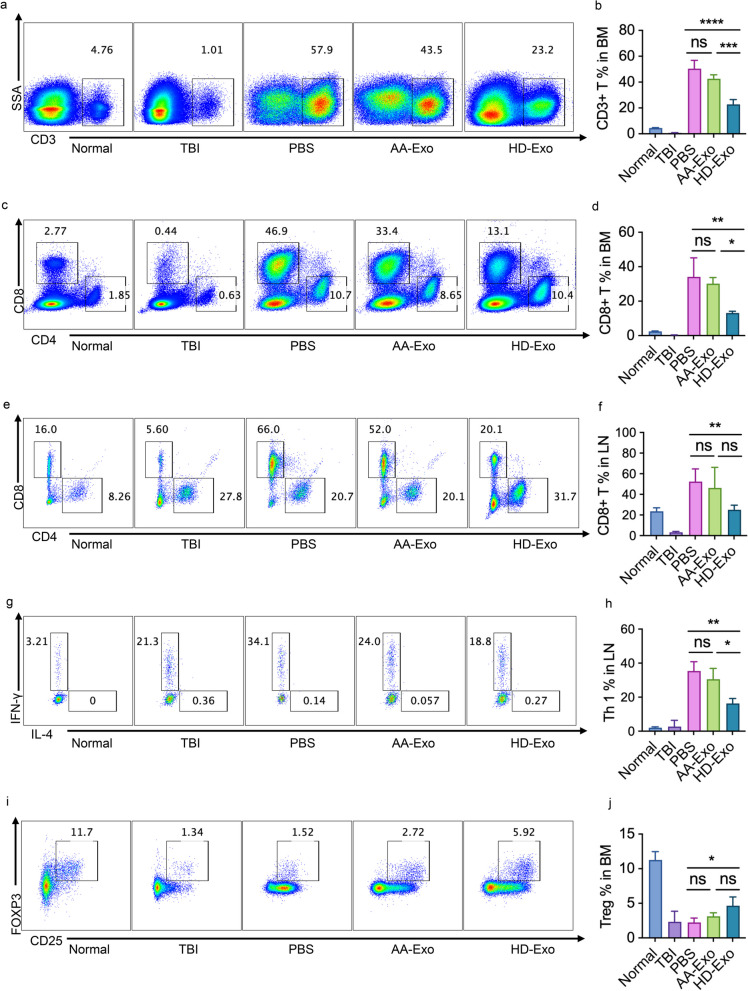
Different immune suppression effect of AA-Exos and HD-Exos in treating AA mice. **a**–**d** AA-Exos showed less potency in reducing the percentage of CD3 + T cells and CD8 + T cells of BM. **e**, **f** HD-Exos showed significant inhibition effect on CD8 + T subsets in LN while AA-Exos did not. **g**, **h** Capacity of CD4 + T cells differentiate to Th1 cells was suppressed intensively in HD-Exo group than AA-Exo group. **i**, **j** AA-Exo group had no significant effect on the preservation of Treg in BM while HD-Exo had. (* for 0.01 ≤ *p* < 0.05; ** for *p* < 0.001; *** for *p* < 0.0001, ns for not significant; *n* = 6)

**Fig. 3:**
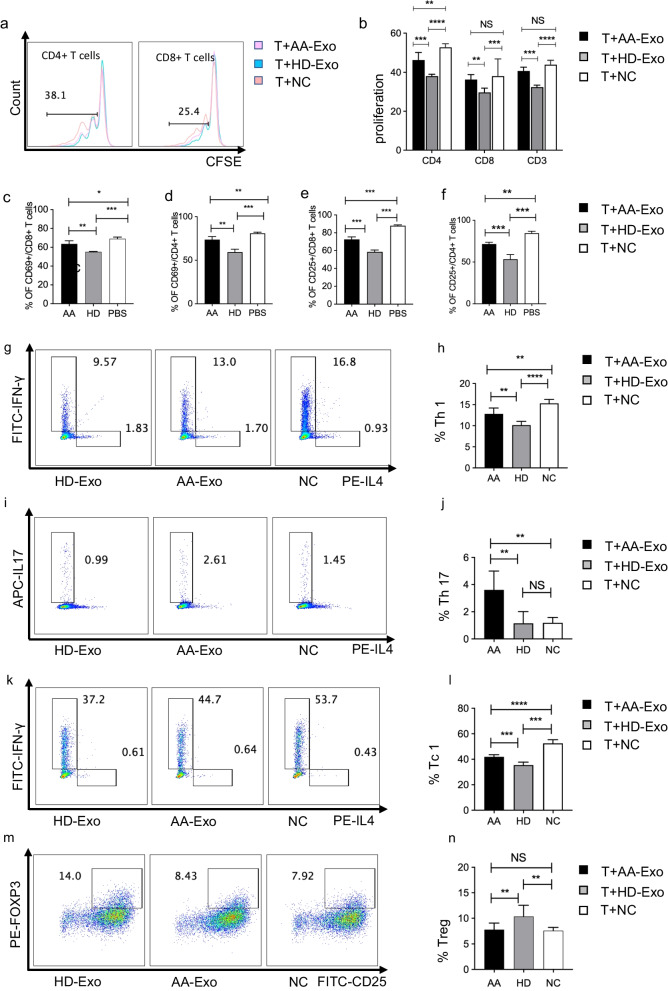
Distinctions in immune regulation functions between AA-Exos and HD-Exos in vitro. **a**, **b** AA-Exos had less suppression effects on proliferation of CD3 + T cells, CD4 + T cells and CD8 + T cells than HD-Exos. **c**–**f** AA-Exos had less suppression effect on CD4 + T and CD8 + T cells activation compared with HD-Exos. **g**, **h** AA-Exos had less suppression effect on CD4 + T cells differentiation to Th1; **i**, **j** AA-Exos had less suppression effect on CD4 + T cells differentiation to Th 17; **k**, **l** AA-Exos had less suppression effect on CD8 + T cells differentiation to Tc1. m, n, AA-Exos had impaired function on inducing Treg. (* for 0.01 ≤ *p* < 0.05; ** for *p* < 0.001; *** for *p* < 0.0001, ns for not significant; *n* = 6)

As shown in Fig. 2a–d, both AA-Exos and HD-Exos reduced the percentage of CD3 + and CD8 + T cells in BM with a more obvious suppressive effect in HD-Exo groups. HD-Exos inhibited CD8 + T cells proportion in LN compared with negative control while AA-Exos did not (Fig. 3e, f). Moreover, HD-Exos treatment suppressed the differentiation of CD4 + T cells into Th1 cells more effectively compared to AA-Exo treatment (Fig. 2g, h).

Tregs played important role in the pathogenesis of AA [16]. Exosomes have been shown to induce Treg generation [17]. Therefore, we examined the changes of proportion of Tregs. Tregs were marked by CD4 + CD25 + FOXP3 + T cells. The population of Treg can hardly be detected in AA mice, but the Treg percentage recovered partially after the administration of HD-Exos, whereas the AA-Exo group did not show a significant effect on preserving Tregs in BM (Fig. 2i, j).

Given above, it suggested that there was systemic alleviation of its excessively immune states in HD-Exo group, while the therapeutic effect of AA-Exos was comparatively weaker.

### Distinctions in immune regulation between AA-Exos and HD-Exos in vitro

To further investigate the mechanism behind the weakened immunosuppressive effect of AA-Exos, as well as to study the effects on T cells thoroughly, we established an in vitro coculture system for a more comprehensive study. CD3 + T cells were sorted by magnetic beads from PBMC and labeled with CFSE for coculturing with AA-Exos or HD-Exos. Fig. 3a, b shows that AA-Exos had less suppression effects on proliferation of CD3 + T cells, CD4 + T cells and CD8 + T cells than HD-Exos. Abnormal activation of T cells was associated with the pathophysiology of AA [1]. After coculturing T cells with exosomes, reduction of T cell activation markers CD25+/CD69+ was observed but AA-Exos had impaired suppression effect compared with HD Exos (Fig. 3c–f, Additional file 2: Figure S2). The differentiation effect of T cells was also affected by exosomes. After stimulating with T cell cocktail for six hours, CD4 + IFNγ + IL-4- T cells (Th1) were detected by flow cytometry. The percentages of Th1 cells in CD4 + T cells in AA-Exos group were 13.0% compared with 9.6% in HD-Exos group, and the ratio of Th1/Th2 was 15.5 vs 9.8 (*p* < 0.05, Fig. 3g,h). The percentages of Th17 cells in CD4 + T cells in AA-Exos group were 2.6% compared with 1.0% in HD-Exos group (*p* < 0.01), (Fig. 3i,j). The percentage of CD8 + T cells different to IFNγ + IL-4-T cells (Tc1) was also inhibited in exosomes treated groups, but the AA-Exos group had higher percentage of Tc1(44.7% vs 37.2%, *p* < 0.001), (Fig. 3k, l). Therefore, we concluded that AA-Exos had less inhibiting function on differentiation than HD-Exos did.

Previous studies disclosed that exosomes enhanced the percentage of Tregs during the culture of PBMC [17]. To investigate whether the induction of Treg was affected, AA-Exos or HD-Exos were added in the culture medium of PBMC with the presence of anti-CD3 and IL-2. After 3-day induction, the percentage of Tregs in AA-Exo group was 7.68% compared with 14.0% in HD-Exo group (Fig. 3m, n), which showed an impact function of AA-Exos in inducing Treg.

Considering all the data above, we concluded that the immune suppression function was impaired in AA-Exos at least to some extent compared with HD-Exos, as well as the Treg-inducing function.

### Critical role of miRNA in the interaction between exosomes and T cells

The RNA cargo of exosomes was believed to be a key mediator exerting their function, especially the carry of miRNA [18], which was considered an important regulatory factor and had multiple functions to affect many pathways at the same time. Hence, microarray analysis was performed on randomly selected HD-Exos and AA-Exos samples to explore the expression profiles of microRNAs.

**Fig. 4:**
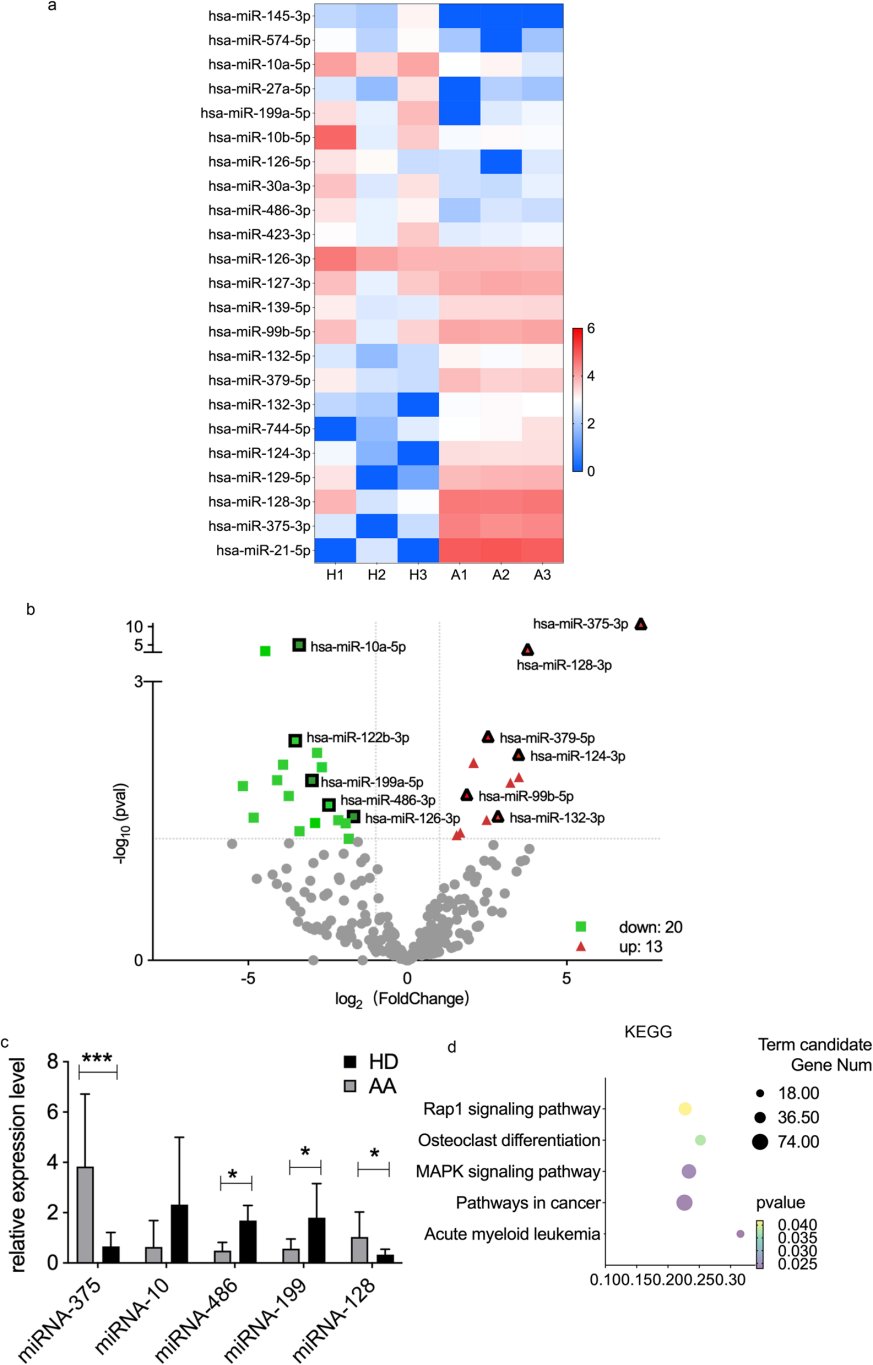
Landscape of miRNA expression profiles in AA-Exos and HD-Exos. **a** Hierarchical cluster analysis of differential miRNAs. **b** Volcano plot showed the differentially expressed miRNAs. **c** Verification of the expression of miRNA by RT-PCR. The bars showed mean ± SD. **d** The KEGG pathway showed the major biochemical metabolic pathways of differentially expressed miRNAs. (* for 0.01 ≤ *p* < 0.05; *** for *p* < 0.001, *n* = 10)

The Pearson correlation heat map calculated by fragments per kilobase per million (FPKM) values of miRNA expression illustrated the relationship between HD and AA groups (Additional file 3, Figure S3a) while the principal component analysis (PCA) of the miRNA expression showed a clear separation between HD and AA in the first principal component (Additional file 3, Figure S3b). The DESeq2 analysis based on negative binomial distribution was adopted for samples because of biological duplication. Hierarchical cluster analysis of differential miRNA was used to exhibit the pattern of differential miRNA expression between the two groups (Fig. 4a). We screen the differentially expressed miRNA under the standard of adjusted *p* < 0.05 &| log2 (foldchange) |> 1.5 with the result displayed in the volcano plot (Fig. 4b), among which 20 miRNAs were down-regulated while 13 were upregulated. Among the most differentially expressed miRNA, hsa-miR-10, hsa-miR-375, hsa-miR-128, hsa-miR-486, hsa-miR-199 draw our attention with their involvement in immune regulation as previously reported [19–21]. We verified the expression level of the 5 miRNAs by RT-PCR. The result showed that hsa-miR-375, hsa-miR-128, hsa-miR-199, hsa-miR-486 had significantly different expression between AA and HD group (*p* = 0.0004, *p* = 0.0414, *p* = 0.0141 and *p* = 0.0217, respectively). The other miRNAs, though not significantly, showed the same tendency with the sequence result (Fig. 4c).

Major biochemical metabolic pathways and signal transduction pathways involved in candidate target genes were explored by KEGG pathway. Plot of KEGG rich clusters of candidate target genes reminded us the involvement of MAPK pathway and Rap1 pathway in the pathophysiology of the disease (Fig. 4d).

### Distinguishable landscape of transcriptome of T cells treated by AA-Exos and HD-Exos

**Fig. 5:**
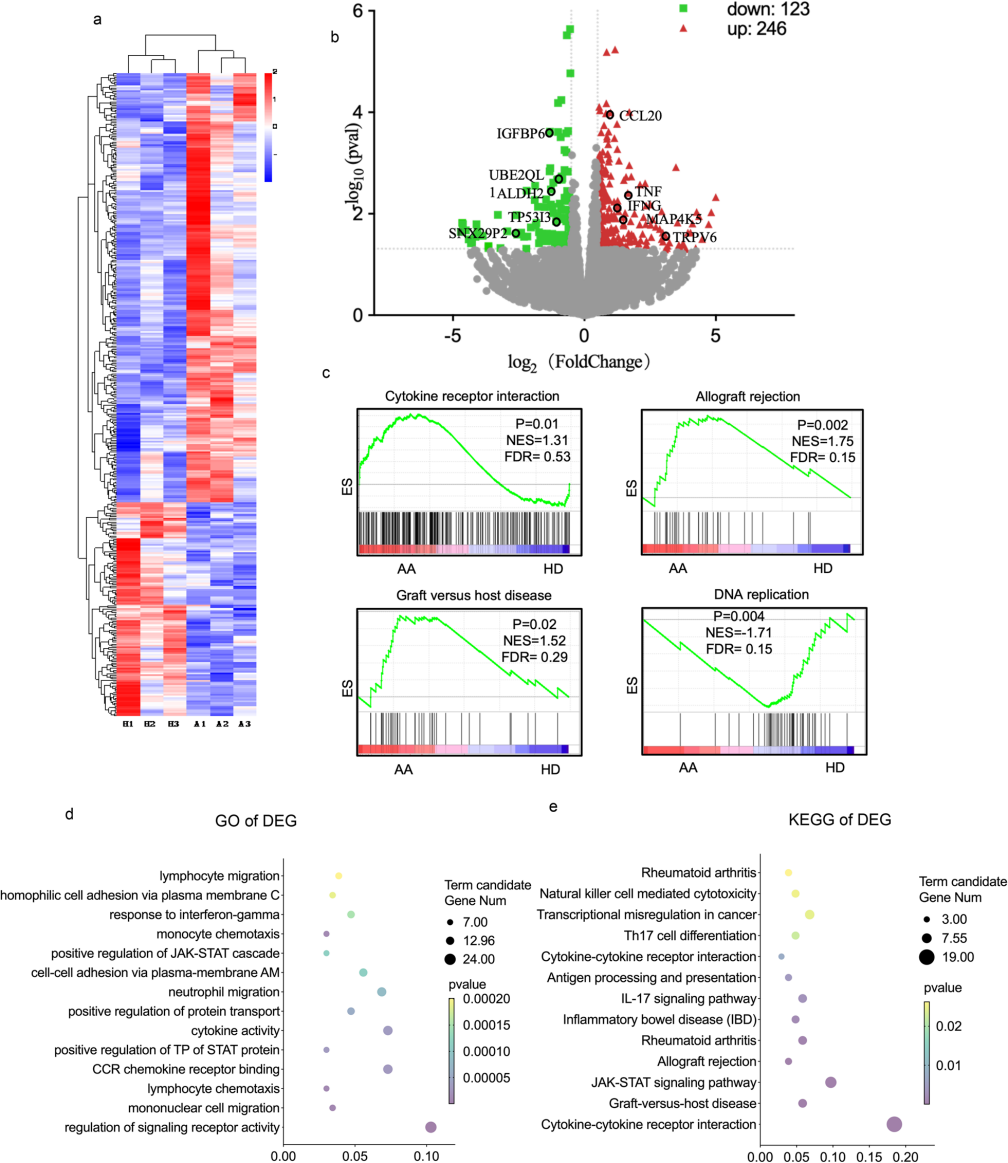
Distinguishable landscape of transcriptome of T cells treated by exosomes derived from AA-MSC and HD-MSC. **a** Unsupervised hierarchical clustering analysis calculated by FPKM values of transcriptome expression exhibit distinguishable patterns between HD and AA groups. **b** The volcano map displayed the differential genes. **c** Gene set enrichment analysis revealed that cytokine receptor interaction, allograft rejection, graft-versus-host disease were enriched in AA-Exo group and DNA replication was enriched in HD-Exo group. **d** The most significant signal pathway of GO enrichment analysis of DEG. **e** The most significant signal pathway collected by KEGG pathway analysis of DEG

The CD3 + T cells were collected from HD and cocultured with exosomes derived from AA-MSC and HD-MSC for 3 days before harvest for genome-wide RNA sequencing (RNA-seq), named AA group and HD group. Correlation coefficients of intra-group and inter-group samples were calculated according to FPKM values of all genes in each sample, and heat maps were drawn to visually display relationship between different samples (Additional file 4, Figure S4a). PCA of the RNA expression unveiled that there were two separated clusters between HD and AA group in sight of the first principal component (Additional file 4, Figure S4b). Differential expression analysis of two groups was performed using DESeq2 to explore the distinguish expression of transcriptome. Unsupervised hierarchical clustering analysis calculated by FPKM values of transcriptome expression exhibited distinguishable patterns between HD and AA groups (Fig. 5a). The volcano map displayed the differential genes compared the experimental group in Fig. 5b. There were 123 genes downregulated while 246 were upregulated with the standard of adjusted *p* < 0.05 &| log2 (foldchange) |> 1.5. Enrichment analysis of differentially expressed genes (DEGs) was carried out for searching the function of distinguish genes. Firstly, GO enrichment analysis of DEGs was implemented with the standard of corrected P value less than 0.05; Fig. 5d displays the most significant 20 terms which were related to CCR chemokine receptor binding, lymphocyte migration, regulation of JAK-STAT cascade, etc. Accordingly, signal pathway related to allograft rejection, Th17 cell differentiation, etc. was collected by KEGG pathway analysis (Fig. 5e). Besides, gene set enrichment analysis also revealed that allograft rejection, cytokine receptor interactions and graft-versus-host disease were enriched in AA-Exos coculturing CD3 + T cells, DNA replication was enriched in HD-Exos coculturing CD3 + T cells (Fig. 5c) which uncovered that the involvement in the dysregulated function of T cell might account for part of the pathogenesis of the disease.

### Network of differentially expressed miRNAs and mRNAs

**Fig. 6:**
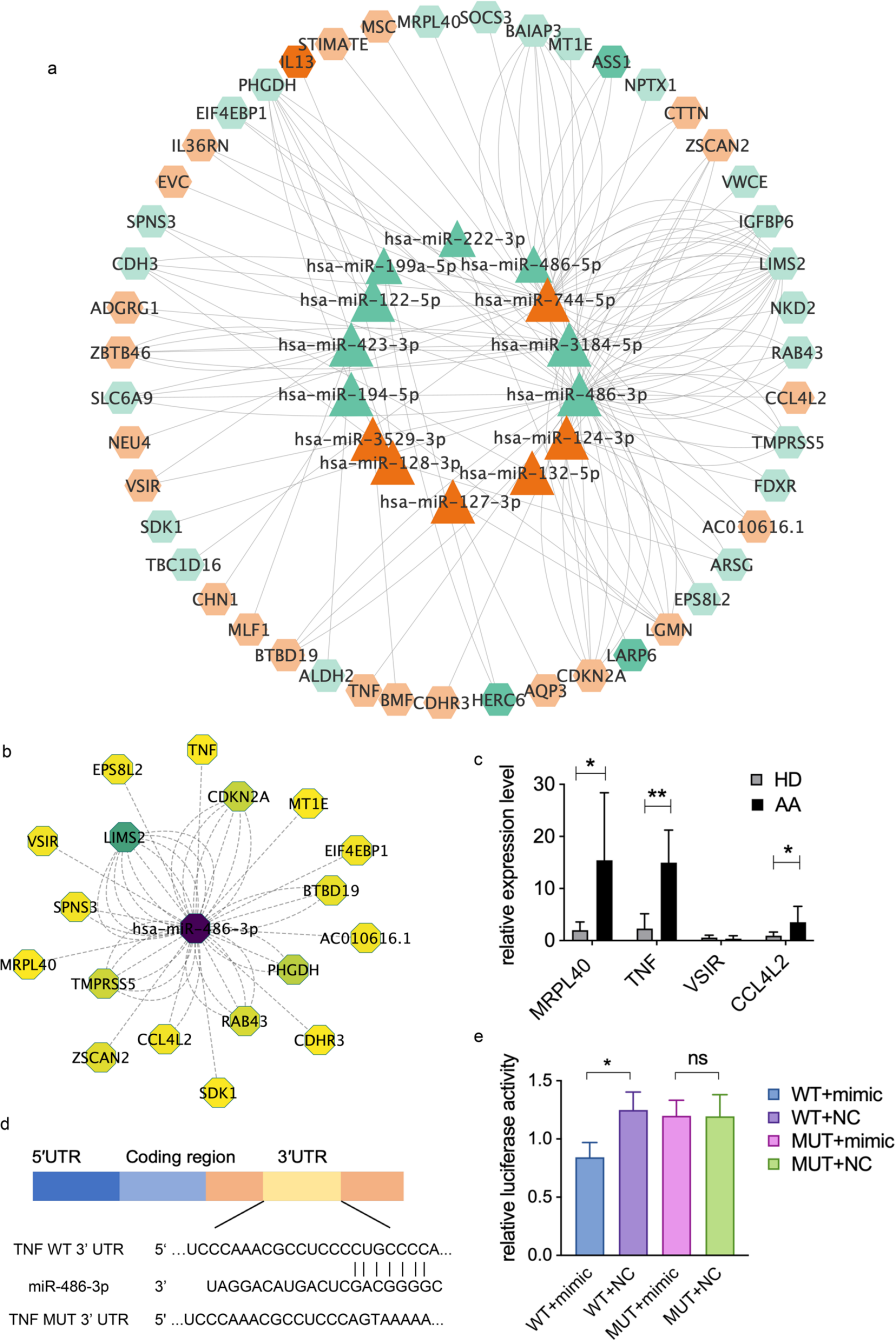
Network of interactions between differentially expressed miRNAs and mRNAs. **a** A miRNA-mRNA network pathway with 61 node and 142 edge including 14 miRNAs and 47 mRNAs, red represented increase of genes or miRNAs, green represented decrease of genes or miRNAs. **b** A miRNA-mRNA network with miRNA-486 as the core node, including its target genes. **c** qPCR validation was performed on DEGs namely MRPL40, TNF, VIIR and CCL4L2. Significant differences in expression were observed in MRPL40, TNF and CCL4L2 (*p* = 0.0116, *p* = 0.0016, *p* = 0.0122, respectively, *n* = 10). **d** The binding sites and mutational sites of TNF 3′UTR to miR-486-3p. **e** MiR-486-3p mimics significantly downregulated the luciferase-based reporter activities transfected with reporter containing WT rather than MUT 3′UTR of TNF mRNA. (* for 0.01 ≤ *p* < 0.05; ns for not significant, *n* = 3)

The miRNAs could inhibit the target gene expression at the post-transcriptional level [9]. Therefore, we performed correlation analysis between upregulated miRNA of exosomes and down-regulated mRNA of T cells, as well as down-regulated miRNA and upregulated mRNA. We construct a miRNA–mRNA network to visually illustrate their interactions. After selecting, a total of 61 node and 142 edge network pathways were constructed, including 14 miRNAs and 47 mRNAs (Fig. 6a). CytoNCA analysis was conducted to identify the most important microRNA based on degree and closeness, which revealed miRNA-486 as a key player. Consequently, a miRNA-mRNA network was constructed using miRNA-486 as the core node, including its targets genes such as VSIR, TMPRSS5, MRPL40, SPNS3, SDK1, BTBD19, TNF and CCL4L2. (Fig. 6b). To validate the findings, qPCR was performed on four DEGs, and significant differences were observed in three of them, namely MRPL40, TNF and CCL4L2 (Fig. 6c, p = 0.0116, *p* = 0.0016, *p* = 0.0122, respectively, *n* = 10). Since TNF plays important role in immune regulation [22, 23], we selected the TNF mRNA to investigate the function of miR-486-3p on its 3' untranslated region. Using TargetScan, we identified a seed sequence (CUGCCCC) in the 3'-UTR of TNF that binds to miR-486-3p (Fig. 6d). Subsequently, dual-luciferase reporter assays were conducted using miR-486-3p mimics and its negative control (NC). The results demonstrated that miR-486-3p significantly downregulated luciferase activity when the reporter contained the wild-type (WT) 3' UTR of TNF mRNA, but not when it contained a mutated (MUT) 3' UTR (Fig. 6e).

In summary, the qPCR validation and dual-luciferase reporter assays provide experimental evidence for the interaction between miR-486-3p and TNF. This network enabled us to better understand the interaction between exosomes and T cells.

## Discussion

Immune-mediated mechanisms may account for 70% patients of acquired aplastic anemia because of the efficiency of immunosuppressive therapy. However, there are still 30% patients remained refractoriness or relapse [24]. The microenvironment of BM may participate in the pathogenesis of AA with contributing to the abnormal immune reaction which may be regarded as potential targets for therapy of inflammatory diseases [25]. MSCs were considered as crucial components in BM microenvironment, and MSC‐Exosomes could partially substitute MSC in its immune regulation function [26]. Additionally, scholars had reported that MSC-EV conditionally ameliorated immune-mediated disease such as GVHD and AA [15, 17]. As MSC‐Exosomes carried different nucleic acids, including miRNAs, it was reasonable to consume those miRNAs were important regulating factors in immune response between different cells [26].

Nonetheless, the role of the exosomes derived from MSC of AA patients in participating the dysregulated immune response was still unknown at molecule level. Our research not only revealed the different expression profile of miRNA carried by exosomes of AA-MSC compared with HD-MSC, but also illustrated the network of the signal transduction between miRNA carried by exosomes and its target mRNA in CD3 + T cells.

It was reported that conditioned exosomes can partially ameliorate immune-mediated bone marrow failure in AA murine model [15]. Consistent with previous report, we found that HD-Exos other than AA-Exos can improve the clinical manifestation of AA mice. Mice in HD-Exo group had shown recovering in RET and HB compared with NC and AA-Exo group. We further compared the immune suppression function of AA-Exos and HD-Exos in vivo. We evaluated the immune states of AA mice and found that the over activated T cell functions were significantly rescued by HD-Exos but not AA-Exos. Given above, the efficiency might be accounted by the alleviation of excessive immune states in HD-Exo group, while the ineffectiveness of AA-Exos treatment can partially be explained by the impaired suppression effect on T cells.

To investigate the immunomodulatory function of MSC‐exosomes thoroughly, CD3 + T cells were cocultured with AA-Exos or HD-Exos. The imbalance of Th1/Th17/Th2 cells and significantly decreasing Tregs may account for the AA pathogenesis [1, 16]. Our findings proved that both AA-Exos and HD-Exos had inhibition effect on CD3 + T cells in terms of cell proliferation, differentiation and activation, but AA-Exos had defect in suppression effects compared with HD-Exos. As regarding the Treg inducing, the percentage was much lower in AA-group so as to produce a weaker immune suppression effect [27]. The imbalance of Th1/Th17/Th2 precent was more obvious in AA-Exos group, so we might speculate that exosomes from AA-MSC formed a deficient immune suppressive environment resulting in an overactive T cell activity.

Previous studies revealed that immune regulation effects of exosomes partially attributed to miRNAs. The miRNA interacted with the 3′ untranslated region of its target gene and influenced the differentiation and proliferation of T cells [20]. We selected differentially expressed miRNAs between AA-Exo and HD-Exo and analyzed the expression profile. We found that many differentially expressed miRNAs were involved in immune response. For example, miR-10 was reported to stabilize Tregs [28, 29] and had a lower expression in autoimmune diabetes mice model [30]; besides, miR-128 could inhibit Treg cells [31]. The result of our in vitro experiment was in accordance with their result that the miR-10a-5p and miR-10b-5p are lower in AA-Exo group while miR-128 was highly expressed with decreasing proportion of Treg cells. The miR-375 was considered associated with the CD8 + T cell-mediated immune responses [32] while we found that it was the biggest different upregulated miRNAs in AA-Exos. The miR‐199a had drawn our attention because previous study [19] revealed that they were downregulated in T cell populations of AA patients, which was the same tendency as we detected. The miRNA-486 has been demonstrated to modulating inflammatory responses [21].

Since the in vitro experiment strongly indicated that there were significant differences in immunoregulation function of AA and HD exosomes, we sequenced transcriptome of CD3 + T cells to further analysis the interaction between miRNA and its target cells on molecular level. By comparison of the transcriptome profiles of T cells in response to AA-Exos and HD-Exos, 246 significantly upregulated and 123 downregulated mRNAs were identified. The GO analysis of the DEGs revealed that they were involved in a series of cellular processes, lymphocyte chemotaxis, lymphocyte migration, response to interferon-gamma, etc. KEGG analysis illustrated that critical pathways associated with T cell function, such as Graft-versus-host disease, Th17 cell differentiation and JAK-STAT signaling pathway, were upregulated after coculturing with AA-Exos. Previous data uncovered the JAK-STAT pathway were associated with anti-apoptosis function in T cells [33]. GVHD usually occurred in an T cell over activated immune environment [34]. Differentiation of Th17 cells, a pro-inflammatory cell type, contributed to many autoimmune diseases, including AA [35]. Collectively, our results demonstrated that AA-Exos may participant in the process of over activating T cells and inducing differentiation towards Th17 cells.

As miRNAs acted to decrease the expression of mRNAs, we further compared the target genes of upregulated miRNA of exosome and down-regulated mRNA of T cells and that of down-regulated miRNA with upregulated mRNA to find profound connection between exosomes and T cells. The overlap of DEGs between miRNA and mRNA was analyzed by GO enrichment with the result highlighted the signal transduction and cytokine receptor binding in biological process. A network including 14 miRNAs and 47 mRNAs was constructed. As the network showed, miRNA usually had more than one target genes while mRNA, in turn, was regulated by multiple miRNAs, resulting in a closely associated miRNA-mRNA network.

We analyzed the network's centrality by cytoNCA plugin in Cytoscape, and the results revealed that hsa-miR-486 ranked first in both degree and eigenvector centrality measures. Previous literature has reported that miR-486 acts as a tumor suppressor, playing a role in various cellular processes such as cell differentiation, proliferation and apoptosis [36]. Moreover, its target genes were also located in central positions within the network. We performed qPCR validation for MRPL40, TNF, VSIR and CCL4L2 with the results in accordance with the sequence data. Besides, dual-luciferase reporter assays provide functional evidence for the interaction between miR-486-3p and TNF mRNA, which play important role in immune signal pathways [22, 23]. Collectively, we speculated that miRNA-486 could target and regulate multiple immune-regulatory genes, potentially playing a crucial role in modulating T cell response.

We are the first to uncover the landscape of differentially expressed miRNAs and explore the relationship between the miRNAs and DEGs of T cells regulated by exosomes through RNA-seq analysis. Still, there is limitation of our research, such as investigations of validation of the miRNA and its target genes in vivo.

## Conclusion

In summary, we have uncovered the miRNA expression profile of AA-Exos and DEGs of exosomes-regulated T cells. Exosomes derive from AA-MSC had an impaired immunosuppressive effect on T cells which may account for part of the pathogenesis of AA as well as provide a new target of AA treatment.

### Supplementary Information

**Additional file 1. Figure S1**. Isolation and characterization of MSC exosomes. a, Schematic diagram of isolation process of the exosomes. b, Flow cytometry of AA-Exos and HD-Exos; there is the no difference of cell markers between AA-Exos and HD-Exos. c, d, Nanoparticle tracking analysis (NTA) of HD-Exos and AA-Exos, respectively; there is the no difference of particle size distribution between AA-Exos and HD-Exos. e, f, Transmission electron microscopy (TEM) of AA and HD exosomes with arrows indicated, both of them showed spherically shape. g, Exosomes were dyed by PKH76 with green with arrows indicated. h, T cells were stained with DAPI. i, Internalization of PKH‐76 green‐labeled exosomes in the cytoplasm of the T cells. Arrows indicated exosomes.

**Additional file 2. Figure S2**. Flow cytometry of activation of T cell cocultured with AA-Exos and HD-Exos. AA-Exos had less suppression effect on CD4+ T and CD8+ T cells activation.

**Additional file 3. Figure S3**. miRNA Expression profile of AA-Exos and HD-Exos a, Pearson correlation heat map of miRNA expression. b, The principal component analysis (PCA) of the miRNA expression.

**Additional file 4.Figure S4**. RNA Expression profile of AA-Exos and HD-Exos. a, Pearson correlation heat map of mRNA expression. b, The principal component analysis (PCA) of the mRNA expression.

**Additional file 5. Table S1**. The characteristics of AA patients and HDs. **Table S2**. Antibodies used in this study. **Table S3**. List of experiments. **Table S4**. Primers used in this study.

## Data Availability

RNA-seq datasets have been uploaded to the Genome Sequence Archive for human (GSA-human No: HRA004360, Project: PRJCA016188). Other data generated or analyzed in this study are included in this published article and its supplementary information files.

## Abbreviations

MSCs: Mesenchymal stem/stromal cells
AA: Aplastic anemia
HD: Healthy donor
BM: Bone marrow
AA-MSCs: AA-derived MSCs
HD-MSCs: HD-derived MSCs
AA-Exos: Exosomes derived from AA-MSCs
HD-Exos: Exosomes derived from HD-derived MSCs
ncRNAs: Noncoding RNAs
IST: Immunosuppressive therapy
PBMNCs: Peripheral blood mononuclear cells
IFN: Interferon
IL: Interleukin
TBI: Total body irradiation
BMMNC: Bone marrow mononuclear cell
H&E: Hematoxylin and eosin
NTA: Nanoparticle tracking analysis
TEM: Transmission electron microscopy
WBC: White blood cells
Hb: Hemoglobin
RBC: Red blood cells
PLT: Platelets
RET: Reticulocyte
FPKM: Fragments per kilobase per million
PCA: Principal component analysis
DEGs: Differentially expressed genes
GO: Gene ontology
KEGG: Kyoto encyclopedia of genes and genomes
DAG: Directed acyclic graph
