# Supplementary figures and images for: Impaired immunosuppressive effect of bone marrow mesenchymal stem cell-derived exosomes on T cells in aplastic anemia
Source: Stem Cell Res Ther. 2023 Oct 4;14:285. doi: 10.1186/s13287-023-03496-0 (PMC10552221; doi:10.1186/s13287-023-03496-0)

c


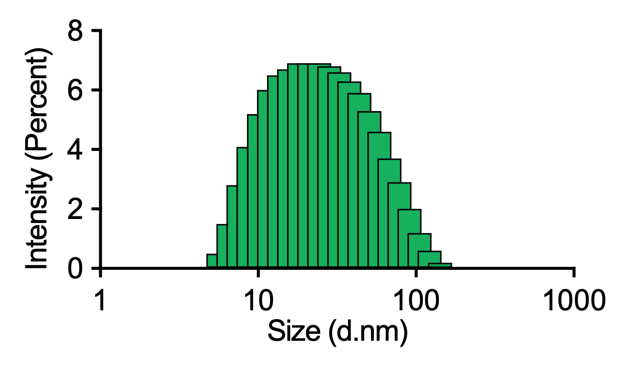


d

e


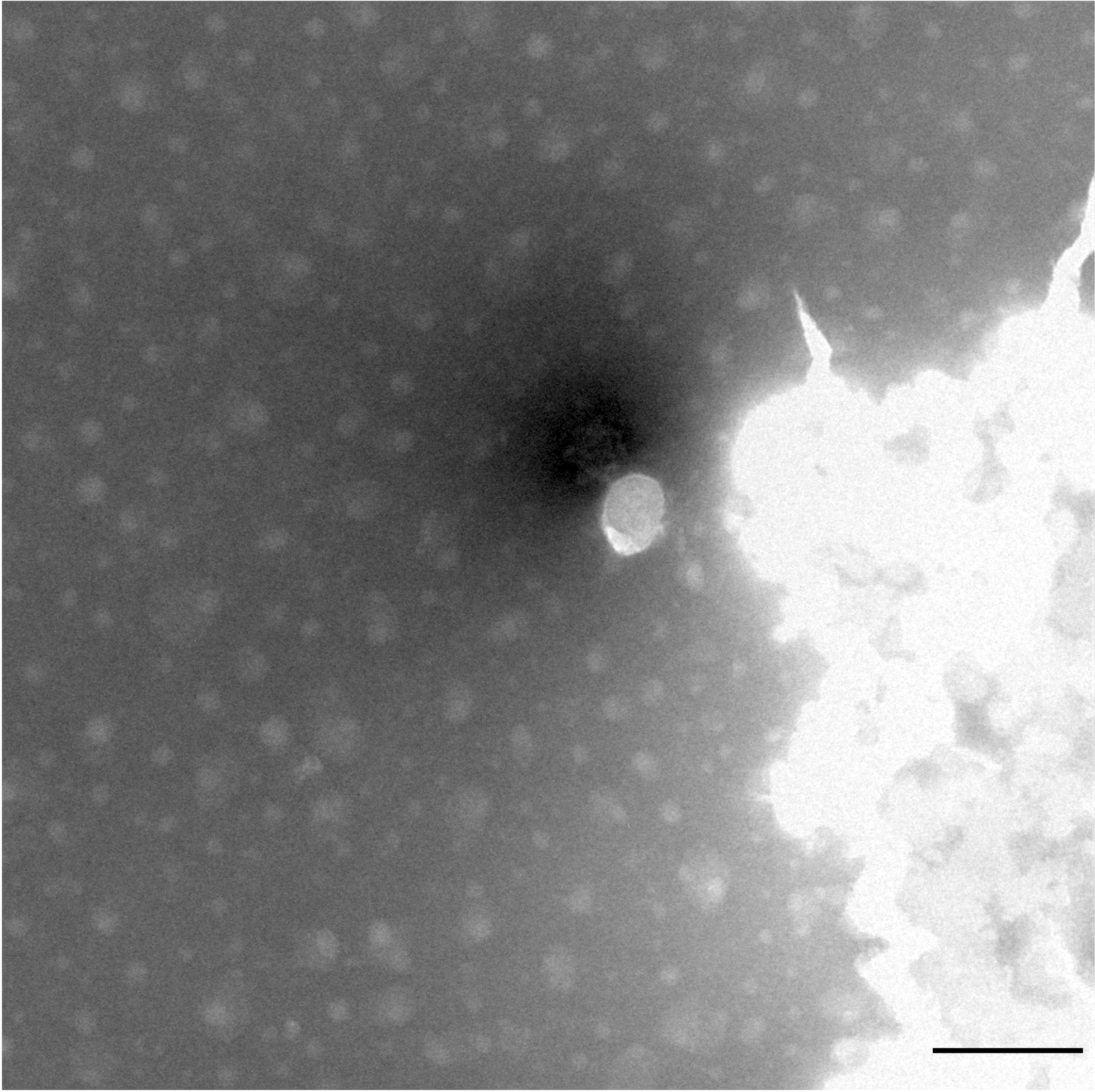


200nm

f

200nm


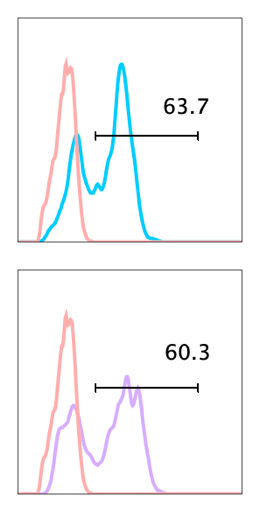

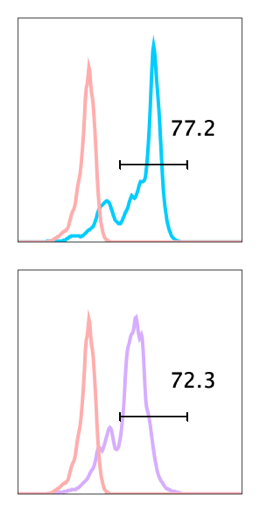


NC

HD-Exo

AA-Exo

b

Count

CD81

CD63


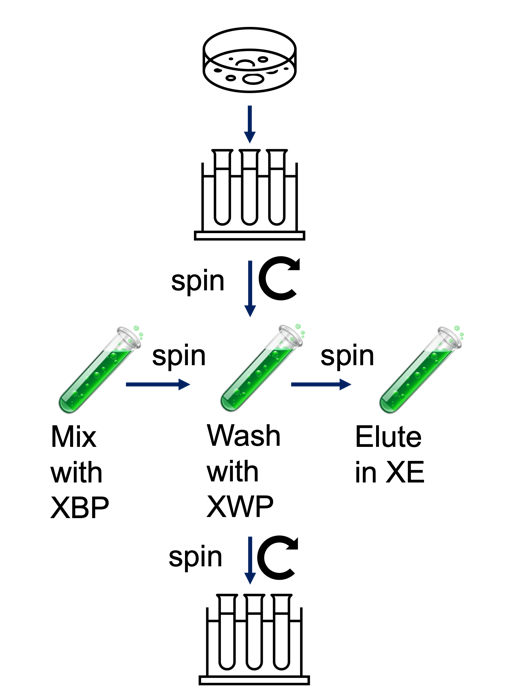


a


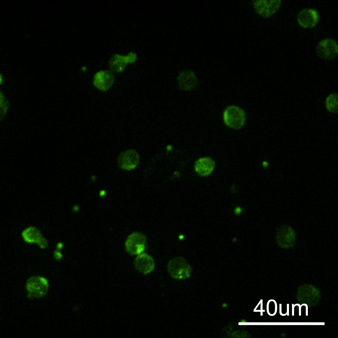

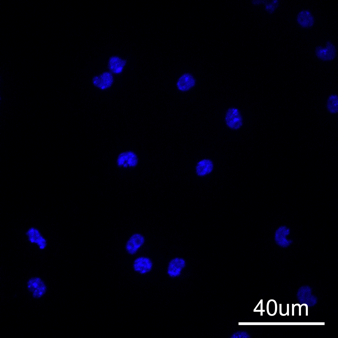

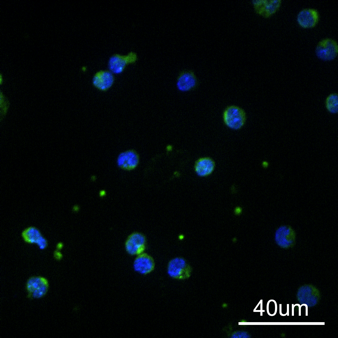


g

h

i

Supplement: Supplementary file 1 — Additional file 1. Figure S1. Isolation and characterization of MSC exosomes. a, Schematic diagram of isolation process of the exosomes. b, Flow cytometry of AA-Exos and HD-Exos; there is the no difference of cell markers between AA-Exos and HD-Exos. c, d, Nanoparticle tracking analysis (NTA) of HD-Exos and AA-Exos, respectively; there is the no difference of particle size distribution between AA-Exos and HD-Exos. e, f, Transmission electron microscopy (TEM) of AA and HD exosomes with arrows indicated, both of them showed spherically shape. g, Exosomes were dyed by PKH76 with green with arrows indicated. h, T cells were stained with DAPI. i, Internalization of PKH‐76 green‐labeled exosomes in the cytoplasm of the T cells. Arrows indicated exosomes. [file 13287_2023_3496_MOESM1_ESM.docx]

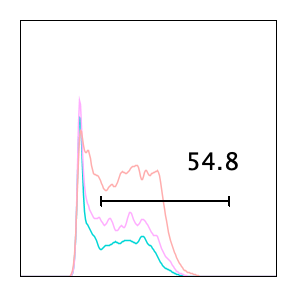


CD4+ T cells


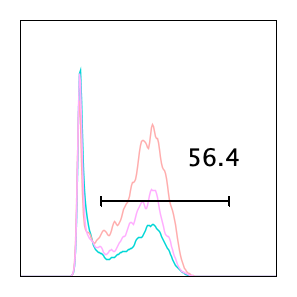


CD8+ T cells


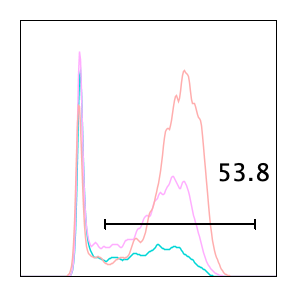


CD4+ T cells


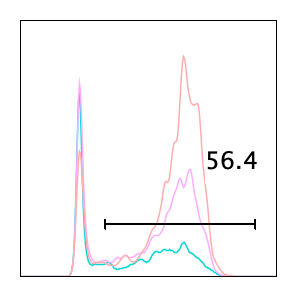


CD8+ T cells

a

Count

CD69

CD25

Count

T+NC

T+AA-Exo

T+HD-Exo

b

Supplement: Supplementary file 2 — Additional file 2. Figure S2. Flow cytometry of activation of T cell cocultured with AA-Exos and HD-Exos. AA-Exos had less suppression effect on CD4+ T and CD8+ T cells activation. [file 13287_2023_3496_MOESM2_ESM.docx]

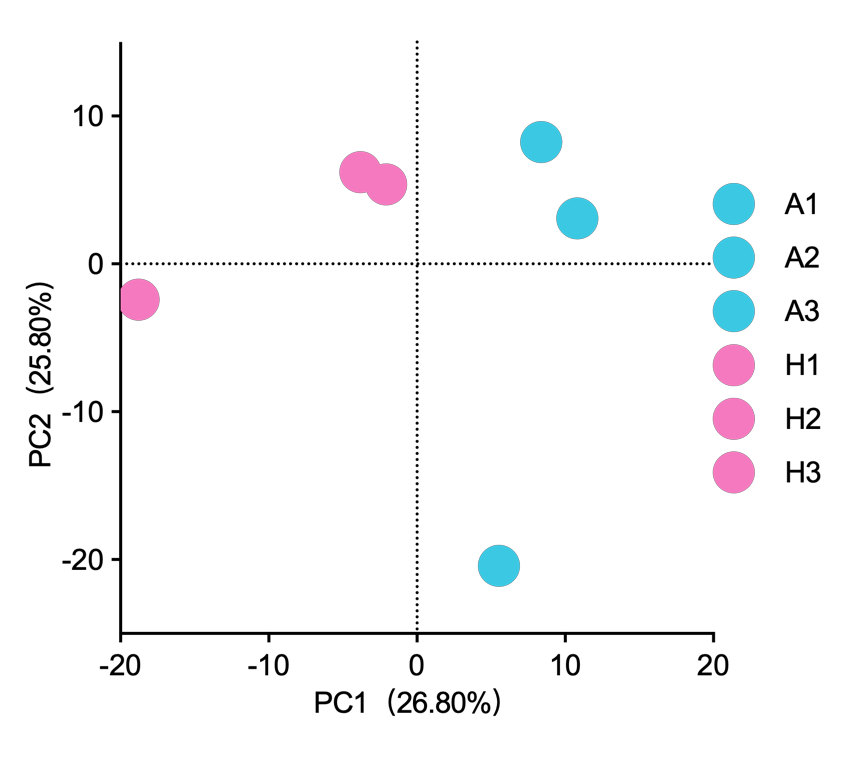


b

a

Supplement: Supplementary file 3 — Additional file 3. Figure S3. miRNA Expression profile of AA-Exos and HD-Exos a, Pearson correlation heat map of miRNA expression. b, The principal component analysis (PCA) of the miRNA expression. [file 13287_2023_3496_MOESM3_ESM.docx]

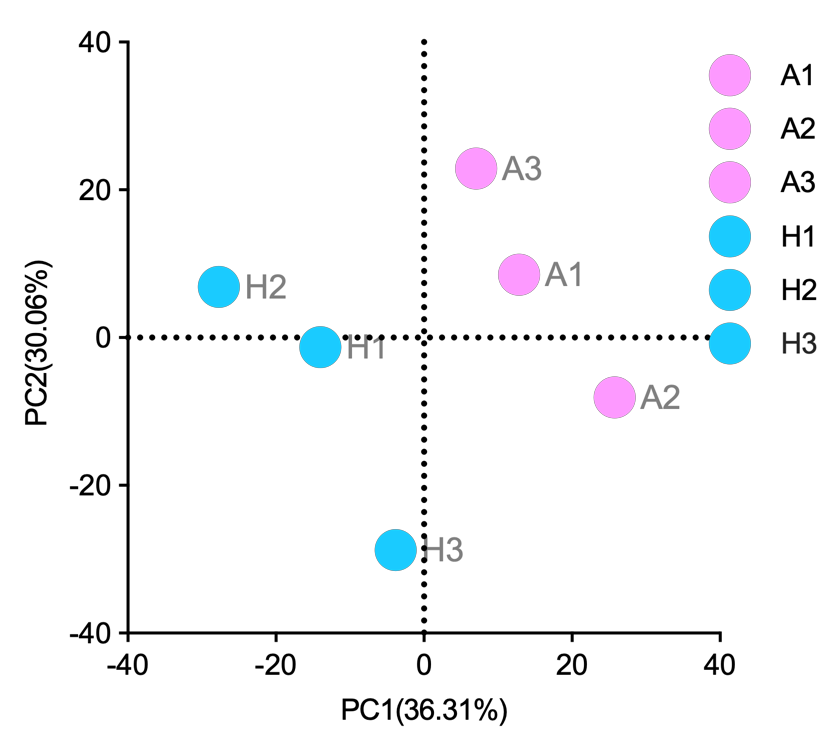

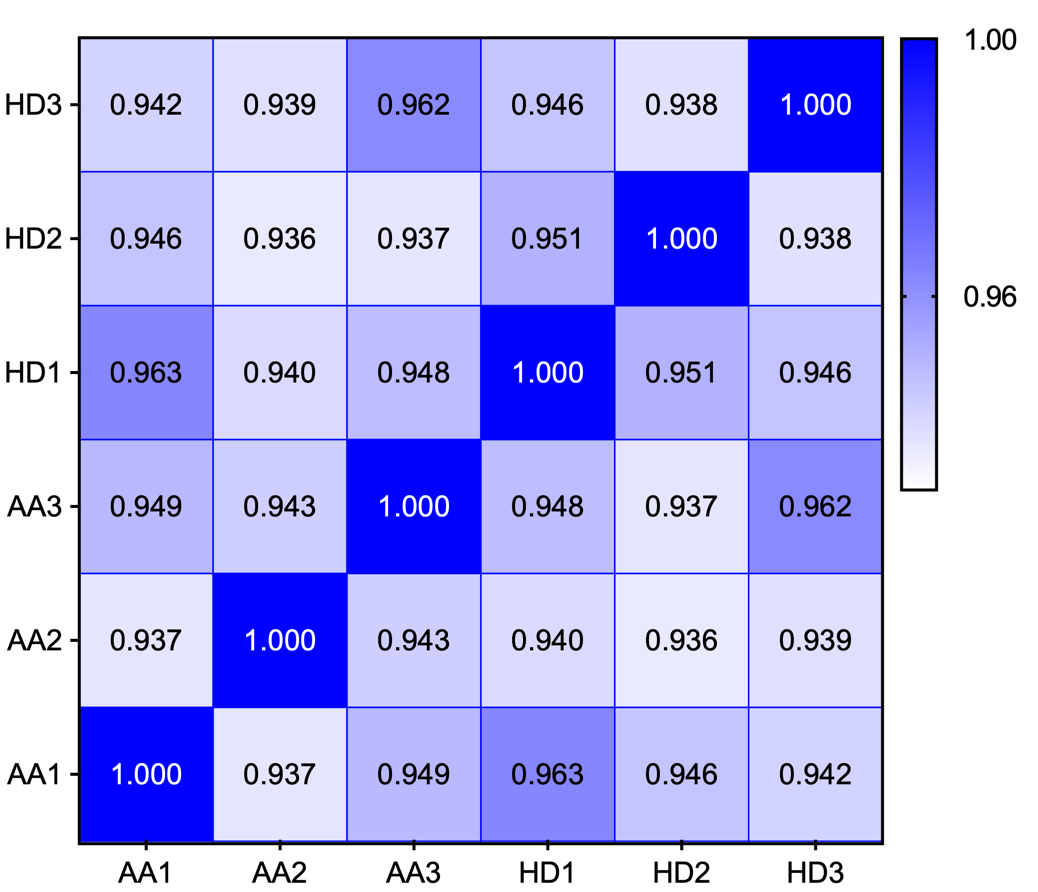


b

a

Supplement: Supplementary file 4 — Additional file 4.Figure S4. RNA Expression profile of AA-Exos and HD-Exos. a, Pearson correlation heat map of mRNA expression. b, The principal component analysis (PCA) of the mRNA expression. [file 13287_2023_3496_MOESM4_ESM.docx]
