# Supplementary material for: Impaired immunosuppressive effect of bone marrow mesenchymal stem cell-derived exosomes on T cells in aplastic anemia
Source: Stem Cell Res Ther. 2023 Oct 4;14:285. doi: 10.1186/s13287-023-03496-0 (PMC10552221; doi:10.1186/s13287-023-03496-0)
Supplement: Supplementary file 5 — Additional file 5. Table S1. The characteristics of AA patients and HDs. Table S2. Antibodies used in this study. Table S3. List of experiments. Table S4. Primers used in this study. [file 13287_2023_3496_MOESM5_ESM.docx]

**Impaired** **Immunosuppressive effect of Bone Marrow Mesenchymal stem cell-derived Exosomes on T cells in Aplastic Anemia**

Shichong Wang1#, Jiali Huo1#, Yilin Liu1, Lingyun Chen1, Xiang Ren1, Xingxin Li 1, Min Wang 1, Peng Jin1, Jinbo Huang1, Neng Nie1, Jing Zhang1, Yingqi Shao1, Meili Ge1* and Yizhou Zheng1*.

1State Key Laboratory of Experimental Hematology, National Clinical Research Center for Blood Diseases, Haihe Laboratory of Cell Ecosystem, Institute of Hematology & Blood Diseases Hospital, Chinese Academy of Medical Sciences & Peking Union Medical College, Tianjin, 300020, China.

#Shichong Wang and Jiali Huo contributed equally.

**Running heads:** Exosomes derived from AA-MSC

*Corresponding authors: Meili Ge, MD, PhD

Diagnostic and Therapeutic Center for Anemic Diseases, State Key Laboratory of Experimental Hematology, National Clinical Research Center for Blood Diseases, Haihe Laboratory of Cell Ecosystem, Institute of Hematology & Blood Diseases Hospital, Chinese Academy of Medical Sciences & Peking Union Medical College, Tianjin, 300020, China.

Tel: 86-022-23909121.

FAX: 86-022-23909093

E-mail: gemeili503@126.com.

*Corresponding authors: Yizhou Zheng, MD, PhD

Diagnostic and Therapeutic Center for Anemic Diseases, State Key Laboratory of Experimental Hematology, National Clinical Research Center for Blood Diseases, Haihe Laboratory of Cell Ecosystem, Institute of Hematology & Blood Diseases Hospital, Chinese Academy of Medical Sciences & Peking Union Medical College, Tianjin, 300020, China.

Tel: 86-022-23909121.

FAX: 86-022-23909093

E-mail: [zheng_yizhou@hotmail.com](mailto:zheng_yizhou@hotmail.com)

**Supplemental figures: figure S1-S4.**

**Figure S1. Isolation and Characterization of MSC Exosomes.**

a, Schematic diagram of isolation process of the exosomes.

b, Flow cytometry of AA-Exos and HD-Exos, there is the no difference of cell markers between AA-Exos and HD-Exos.

c, d, Nanoparticle tracking analysis (NTA) of HD-Exos and AA-Exos respectively, there is the no difference of particle size distribution between AA-Exos and HD-Exos.

e, f, Transmission electron microscopy (TEM) of AA and HD exosomes with arrows indicated, both of them showed spherically shape.

g, Exosomes was dyed by PKH76 with green with arrows indicated. h, T cells was stained with DAPI. i, Internalization of PKH‐76 green‐labeled exosomes in the cytoplasm of the T cells. Arrows indicated exosomes.

**Figure S2.** Flow cytometry of activation of T cell cocultured with AA-Exos and HD-Exos. AA-Exos had less suppression effect on CD4+ T and CD8+ T cells activation.

**Figure S3.** miRNA Expression profile of AA-Exos and HD-Exos

a, Pearson correlation heat map of miRNA expression. b, the principal component analysis (PCA) of the miRNA expression.

**Figure S4.** RNA Expression profile of AA-Exos and HD-Exos

a, Pearson correlation heat map of mRNA expression. b, The principal component analysis (PCA) of the mRNA expression.

**Supplemental Tables: Table S1-S4.**

**Table S1: The characteristics of AA patients and HDs.**

|  | AA(n=35) | HD(n=35) |
| --- | --- | --- |
| Age (Year) | 37.5(8-63) | 32.5(19-55) |
| Gender (Male/Female) | 18/35 | 19/35 |
| Severity (VSAA/SAA) | 14/21 | - |
| WBC(×10^9^/L) | 2.275(0.81-5.55) | 5.97(3.68-9.75) |
| HGB(g/L) | 74(32-145) | 141.5(113-175) |
| PLT (×10^9^/L) | 18.5(1-65) | 248.5(118-394) |
| ANC(×10^9^/L) | 0.365(0.01-2.53) | 3.175(2.07-5.88) |
| ARC(×10^9^/L) | 14.7(1.8-99.7) | 72.55(34.9-148.2) |

*VSAA: very severe aplastic anemia; SAA: severe aplastic anemia; HD: healthy donor; WBC: white blood cell; ANC: absolute neutrophil count; Hb: hemoglobulin; PLT: platelet; ARC: absolute reticulocyte count*

**Table S2. Antibodies used in this study.**

Antibodies for flow cytometry.

| Antibody |  | Cat. NO. | Source |
| --- | --- | --- | --- |
| Anti-human CD73-PE/Cy7 |  | 344009 | BioLegend |
| Anti-human CD90-FITC |  | 555595 | BD Pharmigen |
| Anti-human CD105-APC |  | 800507 | BioLegend |
| Anti-human CD45-APC |  | 560915 | BD Pharmigen |
| Anti-human CD11b-APC/Cy7 |  | 560914 | BD Pharmigen |
| Anti-human CD34-PE/Cy7 |  | 343515 | BioLegend |
| Anti-human HLA-DR-PE |  | 560943 | BD Pharmigen |
| Anti-human CD4-Percy5.5 |  | 300530 | BioLegend |
| Anti-human CD8-APC |  | 301014 | Biolegend |
| Anti-human CD8-PE/Cy7 |  | 344712 | Biolegend |
| Anti-human CD25-PE |  | 302606 | Biolegend |
| Anti-human CD69-FITC |  | 310924 | Biolegend |
| Anti-human IFNγ-FITC |  | 502506 | Biolegend |
| Anti-human IL-4-PE |  | 12-7049-42 | Invitrogen |
| Anti-human IL17A-APC |  | 17-7149-42 | Invitrogen |
| Anti-mouse CD4-FITC |  | 100510 | Biolegend |
| Anti-mouse CD8-Percy5.5 |  | 100734 | Biolegend |
| Anti-mouse IFNγ-APC |  | 505809 | Biolegend |
| Anti-mouse IL-4-PE |  | 504103 | Biolegend |
| Anti-mouse IL-17A-PE/Cy7 |  | 506921 | Biolegend |

**Table S3. List of experiments**

| EXP | Model-strain | DOI | DOT | N | TBI | Treament | note |
| --- | --- | --- | --- | --- | --- | --- | --- |
| 1 | CByB6F1 | 0 | 14 | 12 | no |  | Normal control for experiment |
| 2 | CByB6F1 | 0 | 14 | 20 | 5 Gy |  | TBI group for experiment |
| 3 | C57⇒CByB6F1 | 0 | 14 | 42 | 5 Gy | 200 μl PBS twice a week | negative control group for experiment |
| 4 | C57⇒CByB6F1 | 0 | 14 | 40 | 5 Gy | 200 μl AA-Exos twice a week | AA-Exo group for experiment |
| 5 | C57⇒CByB6F1 | 0 | 14 | 38 | 5 Gy | 200 μl HD-Exos twice a week | HD-Exo group for experiment |

EXP: Experiment number; DOI: Day of initiation; DOT: Day of termination; N: Number of mice.

**Table S4. Primers used in this study.**

| miRNA | qPCR primer |  |
| --- | --- | --- |
| hsa-miR-10a-5p | uacccuguagauccgaauuugug |  |
| hsa-miR-375-3p | uuuguucguucggcucgcguga |  |
| hsa-miR-128-3p | ucacagugaaccggucucuuu |  |
| hsa-miR-199a-5p | cccaguguucagacuaccuguuc |  |
| hsa-miR-486-3p | cggggcagcucaguacaggauu |  |
| mRNA | qPCR primer |  |
| CCL4L2-F | CCGCCTGCTGCTTTTCTTAC |  |
| CCL4L2-R | GCAGACTTGCTTGCCTACCA |  |
| TNF-F | TGTAGCCCATGTTGTAGCAAACC |  |
| TNF-R | TGGTTATCTCTCAGCTCCACG |  |
| MRPL40-F | GAACTCCATCGCCTCCTGTC | |
| MRPL40-R | ATCTTGATGGCAACCTCCCG | |
| VSIR-F | GCGGATGGACAGCAACATTC | |
| VSIR-R | TGACTTTGGCCTCGGGTATC | |
